# Transmesocolic Cystojejunoanastomosis in a Mexican Patient With a Giant Post-acute Pancreatic Pseudocyst Following Acute Pancreatitis: A Case Report

**DOI:** 10.7759/cureus.89228

**Published:** 2025-08-01

**Authors:** Raul A Jimenez-Antonio, Roberto A Alvarado-Hernández, Juan Reyes-Morales, Ludwigvan A Bustamante-Silva

**Affiliations:** 1 General Surgery, Hospital Bicentenario de la Independencia, Institute for Social Security and Services for State Workers (ISSSTE), Tultitlán, MEX; 2 Gastrointestinal Endoscopy, Hospital Bicentenario de la Independencia, Institute for Social Security and Services for State Workers (ISSSTE), Tultitlán, MEX

**Keywords:** acute pancreatitis, endoscopic drainage failure, giant pancreatic pseudocyst, internal drainage, pancreatic fluid collection, surgical management, transmesocolic cystojejunoanastomosis

## Abstract

Giant pancreatic pseudocysts (GPPCs) are a rare but challenging condition, particularly when they reach a size that compromises the surrounding anatomy and the available therapeutic resources. Here, we present the case of a 45-year-old man with a history of severe acute pancreatitis who developed a 5.5-litre cystic collection located in the right hepatorenal space, extending into the infrahepatic retrocolic compartment and displacing the duodenum. This caused significant gastric displacement, progressive abdominal pain, and oral intolerance. Due to the unavailability of endoscopic ultrasound (EUS) and the anatomical unsuitability for cystogastrostomy, caused by marked stomach displacement and lack of safe access, an open surgical approach involving Roux-en-Y (RY) cystojejunoanastomosis via the transmesocolic route was selected. This approach enabled effective dependent drainage without postoperative complications, resulting in a favourable clinical and radiological outcome at one year. This report illustrates the necessity of individualised surgical planning in cases of GPPCs with complex topography. It also underscores the value of transmesocolic cystojejunoanastomosis as a reliable strategy in high-complexity abdominal scenarios where conventional endoscopic options are unavailable or contraindicated.

## Introduction

Pancreatic pseudocysts (PPCs) are a common and clinically significant local complication of acute or chronic pancreatitis. They are defined as encapsulated fluid collections diagnosed four weeks after onset, typically containing pancreatic enzymes, blood, and occasionally non-necrotic tissue, surrounded by an inflammatory fibrous wall that lacks an epithelial lining [1]. PPCs account for between 6% and 16% of pancreatitis cases and up to 80% of cystic pancreatic lesions. Although usually benign, it is estimated that 15-30% of patients develop symptomatic, persistent, or large-volume collections requiring specialised intervention [2].

A giant pancreatic pseudocyst (GPPC) is defined as any encapsulated collection exceeding 10 cm in diameter, with the potential to compress neighbouring organs, impair gastric emptying, cause persistent abdominal pain, or generate obstructive phenomena [3]. These lesions require thorough anatomical and functional evaluation through imaging studies, such as abdominal ultrasound (US), computed tomography (CT), magnetic resonance imaging (MRI), or endoscopic ultrasound (EUS), to determine their location, contents, and anatomical relationships with adjacent vascular and digestive structures [4,5].

The therapeutic approach to PPCs has shifted towards minimally invasive strategies. Endoscopic US-guided drainage has proven highly effective in treating collections adjacent to the gastrointestinal tract that are confined to a single cavity. Its clinical and technical success rates are similar to those of surgical treatment, with shorter hospital stays and lower costs [6]. However, surgery remains the definitive approach for patients with giant pseudocysts [3], atypical locations [7], or significant anatomical displacement [8]. In such cases, Roux-en-Y (RY) cystojejunoanastomosis is a safe and effective alternative, as it allows dependent drainage even when the stomach is displaced, reducing the risk of gastric contamination and haemorrhagic complications [8].

Acute pancreatitis is a growing cause of hospitalisation in Mexico, with an estimated prevalence of 22.4 cases per 100,000 people and a sustained increase over the past two decades [9]. Recent national data show that over 50% of patients are male and under 50 years of age, with gallstones as the primary cause (44.6%), followed by alcohol use (18.5%) and dyslipidaemia, particularly in the southern and central regions of the country [9]. Furthermore, Vázquez-Frías et al. report that up to 12% of cases develop local complications, with PPCs being among the most frequent [10]. There is significant underreporting of this condition in general hospitals where access to EUS is limited. This highlights the importance of improving diagnostic capabilities and ensuring timely surgical treatment in second-level hospitals [10].

## Case presentation

A 45-year-old male patient (BMI: 27.3 kg/m²), with no significant comorbidities and a history of severe acute biliary pancreatitis diagnosed six months prior to his current admission, was managed conservatively during the initial episode with a hospital stay of 10 days. He was admitted to our attention with a four-week history of intermittent epigastric abdominal pain, postprandial fullness, and progressively worsening intolerance to oral intake, with symptoms markedly intensifying during the past 24 hours. The patient was not taking any regular medications at the time of admission.

The initial episode of severe acute pancreatitis was triggered by gallstone migration, with no prior history of alcohol consumption, hypertriglyceridaemia, or other precipitating factors.

On physical examination, his abdomen was distended and tender to deep palpation in the epigastric and right flank regions. There were no signs of peritoneal irritation. Vital signs were within normal limits. The haematology and biochemistry results are shown in Table 1, indicating normal pancreatic function and negative tumour markers.

**Table 1:** Laboratory studies at admission AST: aspartate aminotransferase; SGOT: serum glutamic-oxaloacetic transaminase; ALT: alanine aminotransferase; SGPT: serum glutamic-pyruvic transaminase

Parameter	Patient Value	Reference Range
Leucocytes	10,500/μL	4,000–10,000/μL
Haemoglobin	11.6 g/dL	13.5–17.5 g/dL
Platelets	322,000/μL	150,000–400,000/μL
Amylase	75 U/L	30–110 U/L
Lipase	60 U/L	23–300 U/L
Total Bilirubin	0.31 mg/dL	0.3–1.2 mg/dL
GGT (Gamma-Glutamyl Transferase)	70 U/L	< 55 U/L (age-dependent)
AST (SGOT)	56 U/L	5–40 U/L
ALT (SGPT)	64 U/L	7–56 U/L
Alkaline Phosphatase	131 U/L	44–147 U/L
Creatinine	0.9 mg/dL	0.6–1.3 mg/dL
Carcinoembryonic Antigen (CEA)	1.6 ng/mL	< 5.0 ng/mL
CA 19-9	2 U/mL	< 37 U/mL
Alpha-Fetoprotein	1.3 ng/mL	< 10 ng/mL

An initial abdominal US was performed, which identified a large anechoic cystic structure in the upper right quadrant but was insufficient to fully delineate its size, extension, and relation to adjacent organs. Therefore, a contrast-enhanced CT scan was subsequently requested for a more comprehensive evaluation.

**Figure 1:**
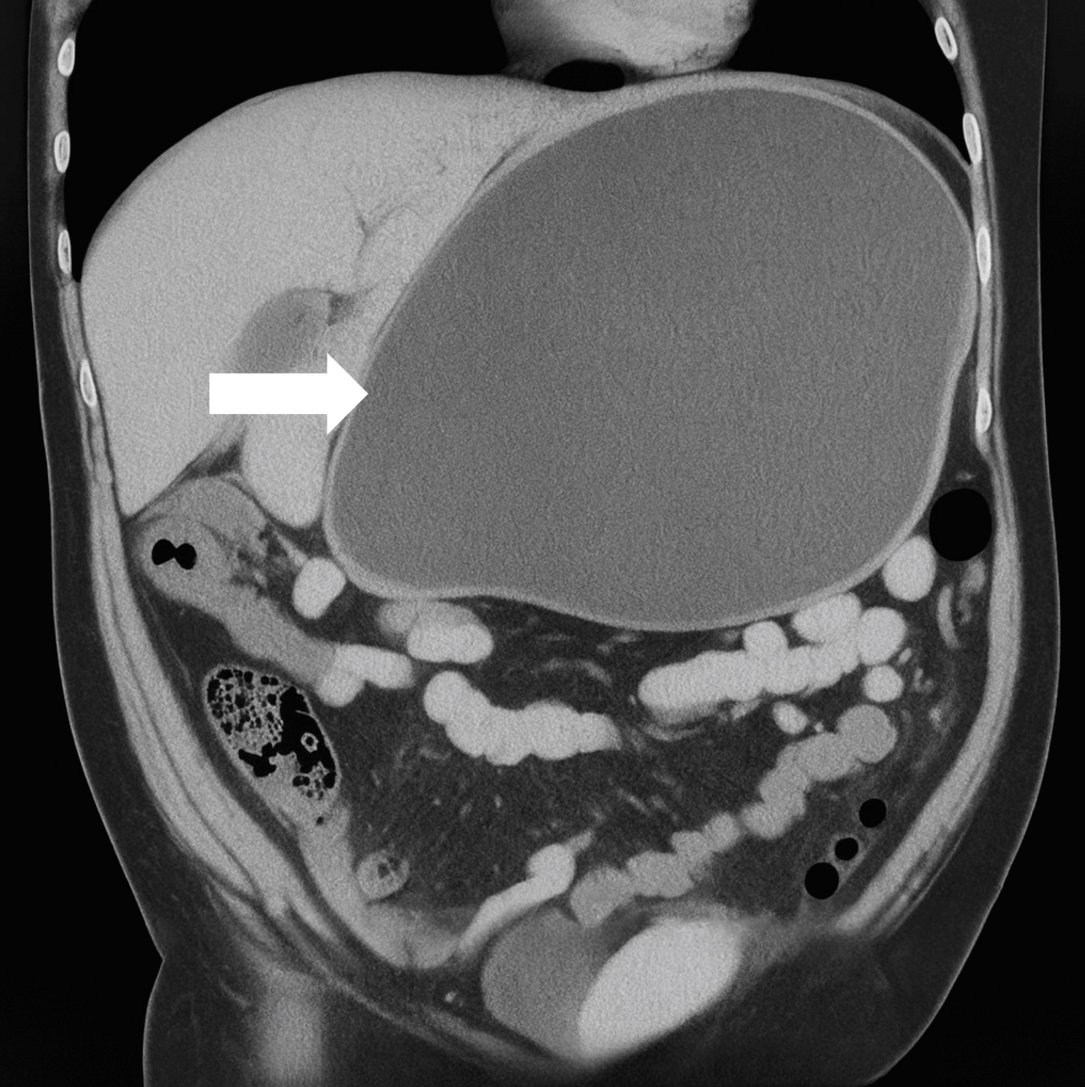
Coronal reconstruction of a simple abdominal tomogram in the non-contrast phase. The tomographic image shows a well-delimited, homogeneous, cystic collection with hypodense content and no internal septations. This collection occupies a large part of the right upper hemiabdomen. The stomach is displaced cephalically, and the small intestine is compressed, suggesting a significant mass effect due to increased intra-abdominal volume.

The CT scan revealed a well-encapsulated cystic lesion measuring 277 × 153 × 223 mm, with an estimated volume of 5,103.5 cc. The lesion was located in the right hepatorenal space, an anatomical region between the inferior surface of the liver and the anterior aspect of the right kidney, also known as Morrison's pouch. It was found to be displacing the stomach superiorly and compressing neighbouring intestinal structures (Figure 1). There were no signs of necrosis, haemorrhage, or internal septations.

Given the lesion's size, mass effect, and the patient's worsening clinical picture, an exploratory laparotomy was indicated. Although CT-guided percutaneous drainage was considered, it was not feasible in our institution at the time of admission due to limited interventional radiology resources. Additionally, the cyst's large volume, thin wall, and absence of internal septations raised concerns about incomplete drainage or recurrence with a percutaneous approach.

The diagnosis of the lesion as a PPC was established through clinical history (severe acute pancreatitis six months prior), biochemical markers (normal amylase/lipase levels, and negative tumour markers), and imaging features: a homogeneous, unilocular, well-encapsulated, non-enhancing cystic structure without mural nodules or septa.

**Figure 2:**
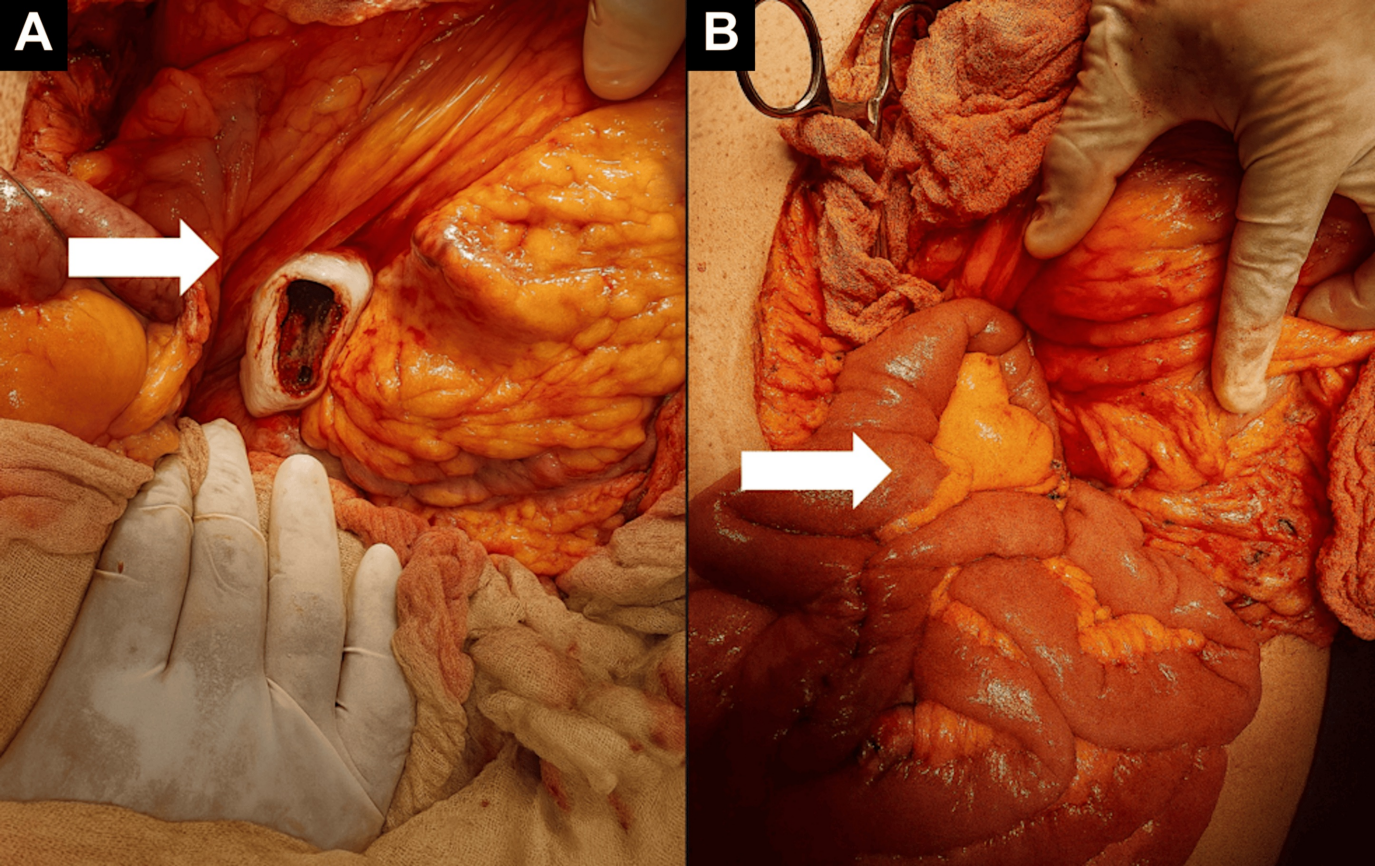
Intraoperative surgical approach to a giant pancreatic pseudocyst. (A) View of the surgical field after opening the transverse mesocolon, showing the pseudocyst capsule, which is widely distended after the liquid contents have been evacuated. The lower border of the stomach appears to be displaced cephalad, which makes transgastric access unsafe. (B) The exposure of the jejunal loop is performed in preparation for a laterolateral Roux-en-Y cystojejunoanastomosis. The adequate mobilisation and orientation of the jejunum towards the region of the pseudocyst can be seen, ensuring dependent, tension-free drainage.

Intraoperatively, a tense, well-defined cystic collection was found. Through controlled puncture, 5.5 litres of clear fluid were evacuated (Figure 2A). Due to superior displacement of the stomach and lack of a safe window for cystogastrostomy, a laterolateral RY cystojejunoanastomosis was performed using a transmesocolic approach. The anastomosis was created using double-layer hand-sewn sutures: an inner full-thickness continuous absorbable suture (polydioxanone 3-0) and an outer seromuscular interrupted suture (silk 3-0). The Blumgart technique was not used. To minimise the risk of cystojejunal suture failure, careful haemostasis, tension-free alignment, and adequate drainage positioning were ensured. No intraoperative or early postoperative complications occurred (Figure 2B).

The patient presented with a satisfactory postoperative evolution, demonstrating adequate tolerance to clear liquids by 24 hours and progressing to a soft diet without nausea or distension. The surgical drain was removed on the second day, after observing scarce serous debit and negative cultures. At 48 hours, laboratory studies revealed hepatic, pancreatic, and haematological profiles within physiological limits, with no signs of septic complications or bleeding. He was discharged on the third day with outpatient follow-up. At the one-month follow-up visit, he remained asymptomatic, with a normal physical examination and no clinical or biochemical evidence of recurrence (Table 2).

**Table 2:** Postoperative control laboratories (48 hours and one month) AST: aspartate aminotransferase; SGOT: serum glutamic-oxaloacetic transaminase; ALT: alanine aminotransferase; SGPT: serum glutamic-pyruvic transaminase

Parameter	48 h post-op	1 month post-op	Reference Range
Leucocytes	8,700/μL	6,400/μL	4,000–10,000/μL
Haemoglobin	11.2 g/dL	12.6 g/dL	13.5–17.5 g/dL
Platelets	285,000/μL	312,000/μL	150,000–400,000/μL
Amylase	65 U/L	52 U/L	30–110 U/L
Lipase	55 U/L	48 U/L	23–300 U/L
Total Bilirubin	0.43 mg/dL	0.41 mg/dL	0.3–1.2 mg/dL
GGT (Gamma-Glutamyl Transferase)	62 U/L	45 U/L	< 55 U/L
AST (SGOT)	40 U/L	31 U/L	5–40 U/L
ALT (SGPT)	44 U/L	38 U/L	7–56 U/L
ALP (Alkaline Phosphatase)	120 U/L	112 U/L	44–147 U/L
Creatinine	0.87 mg/dL	0.91 mg/dL	0.6–1.3 mg/dL
CRP (C-Reactive Protein)	3.2 mg/L	<1 mg/L	< 5.0 mg/L
Procalcitonin	<0.05 ng/mL	<0.05 ng/mL	< 0.1 ng/mL

## Discussion

The management of GPPCs remains a clinical and surgical challenge, particularly in settings where advanced endoscopic resources are unavailable or technically infeasible. In this case, performing a transmesocolic cystojejunoanastomosis enabled effective internal drainage, resulting in complete resolution of symptoms and no recurrence at the one-year follow-up. These findings coincide with the favourable outcomes reported for this technique in specialised surgical series [11].

Although EUS-guided drainage has demonstrated high technical and clinical success rates, its usefulness in GPPCs may be limited by the size, location, and anatomical relationship of the condition to the gastrointestinal tract. Udeshika et al. documented that, even with endoscopic drainage, the management of large collections may necessitate multiple interventions, such as the use of luminal apposing metal stents (LAMS) and additional drains, alongside close imaging and endoscopic follow-up [13].

Similarly, Bhakta et al. emphasise that the surgical approach should be tailored to each patient, bearing in mind the risk of complications, such as bleeding or infection, associated with cystogastrostomy in large pseudocysts [12]. They advocate cystojejunoanastomosis when dependent drainage is deemed safer. This view is supported by Law et al., who reported a higher risk of postoperative complications with direct approaches to the stomach, particularly in cases involving heterogeneous contents or atypical locations [7].

From an anatomical and technical perspective, RY jejunal anastomosis offers several advantages, including a lower risk of gastric contamination and the possibility of dependent bypass. It also has a low recurrence rate, particularly in large pseudocysts exceeding 15 cm in diameter [7,13]. In our case, the absence of early postoperative complications was attributed to the meticulous surgical technique, including careful suture handling, tension-free alignment, and appropriate drain placement. Potential late complications such as anastomotic leakage or recurrence were monitored through clinical and biochemical follow-up, with no adverse events recorded during the first postoperative year.

Additional reports have described the use of transmesocolic RY cystojejunoanastomosis in giant pseudocysts with similarly favourable outcomes and minimal complication rates. These findings reinforce its role as a viable alternative when endoscopic options are limited or contraindicated. Future studies with larger case series may help standardise technical aspects and refine patient selection criteria for this approach.

## Conclusions

The present case demonstrates that transmesocolic cystojejunoanastomosis represents a safe and effective alternative for the management of GPPCs in symptomatic patients, especially in contexts where endoscopic drainage is not feasible. The choice of surgical approach was based on anatomical and clinical criteria, resulting in complete resolution of symptomatology without recurrence at one year. This finding supports what has been reported in the international literature and highlights the need to adapt therapeutic strategies to the care setting, always guaranteeing the safety and effectiveness of individualised treatment.
